# The novel ATM inhibitor (AZ31) enhances antitumor activity in patient derived xenografts that are resistant to irinotecan monotherapy

**DOI:** 10.18632/oncotarget.22920

**Published:** 2017-12-05

**Authors:** Justin Greene, Anna Nguyen, Stacey M. Bagby, Gemma N. Jones, WM. Tai, Kevin S. Quackenbush, Anna Schreiber, Wells A. Messersmith, Kalpana M. Devaraj, Patrick Blatchford, S. Gail Eckhardt, Elaine B. Cadogan, Gareth D. Hughes, Aaron Smith, Todd M. Pitts, John J. Arcaroli

**Affiliations:** ^1^ Division of Medical Oncology, University of Colorado Anschutz Medical Campus and University of Colorado Cancer Center, Aurora, CO, USA; ^2^ Pathology Department, University of Colorado Anschutz Medical Campus, Aurora, CO, USA; ^3^ Division of Medical Oncology, National Cancer Centre Singapore, Singapore; ^4^ Innovative Medicines and Early Development, Oncology, AstraZeneca, Cambridge, UK

**Keywords:** ATM, DNA damage, IRN, PDTX, CRC

## Abstract

Irinotecan, a standard of care therapy for CRC, elicits cytotoxic effects by generating double strand breaks resulting in DNA damage. The activation of the ATM pathway plays a fundamental role in regulating the cellular response and repair to DNA damage. The objective of this preclinical study was to determine whether ATM inhibition would enhance sensitivity to irinotecan treatment. Treatment effects of AZ31, irinotecan or AZ31 + irinotecan were investigated in CRC cell lines and CRC patient derived xenografts. Activation of ATM and downstream targets p-RAD50 and p-H2AX were evaluated by immunohistochemistry. Combinational effects were demonstrated in 4 out of 8 CRC explants. Interestingly, each of the combinational sensitive CRC PDX models were shown to be more resistant to irinotecan single agent therapy. Treatment with irinotecan significantly elevated the ATM pathway evident by an increase in the activation of H2AX and RAD50. Combinational therapy reduced the activation of H2AX and RAD50 when compared to irinotecan alone in the combination sensitive CRC098. AZ31 + irinotecan was effective at reducing tumor growth in tumors that exhibited resistance to irinotecan in our CRC PDX model. These findings support further investigation of this combinational therapy for the treatment of CRC patients.

## INTRODUCTION

Colorectal cancer (CRC) continues to be one of the most common cancers and leading causes of cancer related deaths in the United States [1]. Although significant progress has been made in early detection resulting in higher cure rates, metastatic colorectal cancer (CRC) patients continue to have a poor prognosis. Over the past 20 years, the addition of chemotherapeutic regimens and biologics has incrementally increased the median overall survival in mCRC [2]. Despite these advances, treatment resistance is a major obstacle in significantly impacting the overall survival in these patients. Therefore, novel treatment strategies are needed to overcome treatment resistance and improve overall outcomes.

Irinotecan is a topoisomerase I inhibitor that is used as a standard therapy for the treatment of CRC [3, 4]. Irinotecan exerts its cellular toxicity by generating lethal double-strand DNA breaks resulting in S phase arrest and an induction of apoptosis [5–7]. As a mechanism to overcome the cytotoxic effects of irinotecan, tumor cells trigger several different checkpoint pathways to ensure repair of DNA and ultimately cell survival [8, 9]. One of the major checkpoints activated by double strand DNA breaks is the serine/threonine kinase, ataxia-telangiectasia mutated (ATM) [8–11].

The cellular response to DNA damage is a multifaceted process whereby the MRN complex comprised of MRE11, RAD50 and NBS1 recognize double strand breaks recruiting ATM to the site [12, 13]. ATM interacts with NBS1 and its subsequent activation leads to the phosphorylation of H2AX (Ser139), a histone 2A family protein. H2AX activation facilitates the binding of other effector proteins leading to the phosphorylation of p53 and CHK2 by ATM [8–10, 14]. As a result of chemotherapeutic mediated DNA damage, a coordinated response led by ATM plays a major role in facilitating the cellular repair of DSBs and survival.

Considering the importance of DNA damage checkpoint pathways in mediating cell survival in response to chemotherapeutic DNA damaging agents, in this preclinical study, we set out to determine whether inhibiting the ATM pathway with a novel ATM inhibitor (AZ31) in combination with irinotecan would enhance the cytotoxic effects leading to significant antitumor responses.

## RESULTS

### Enhanced antitumor effects are exhibited by AZ31 in combination with SN38

**Figure 1:**
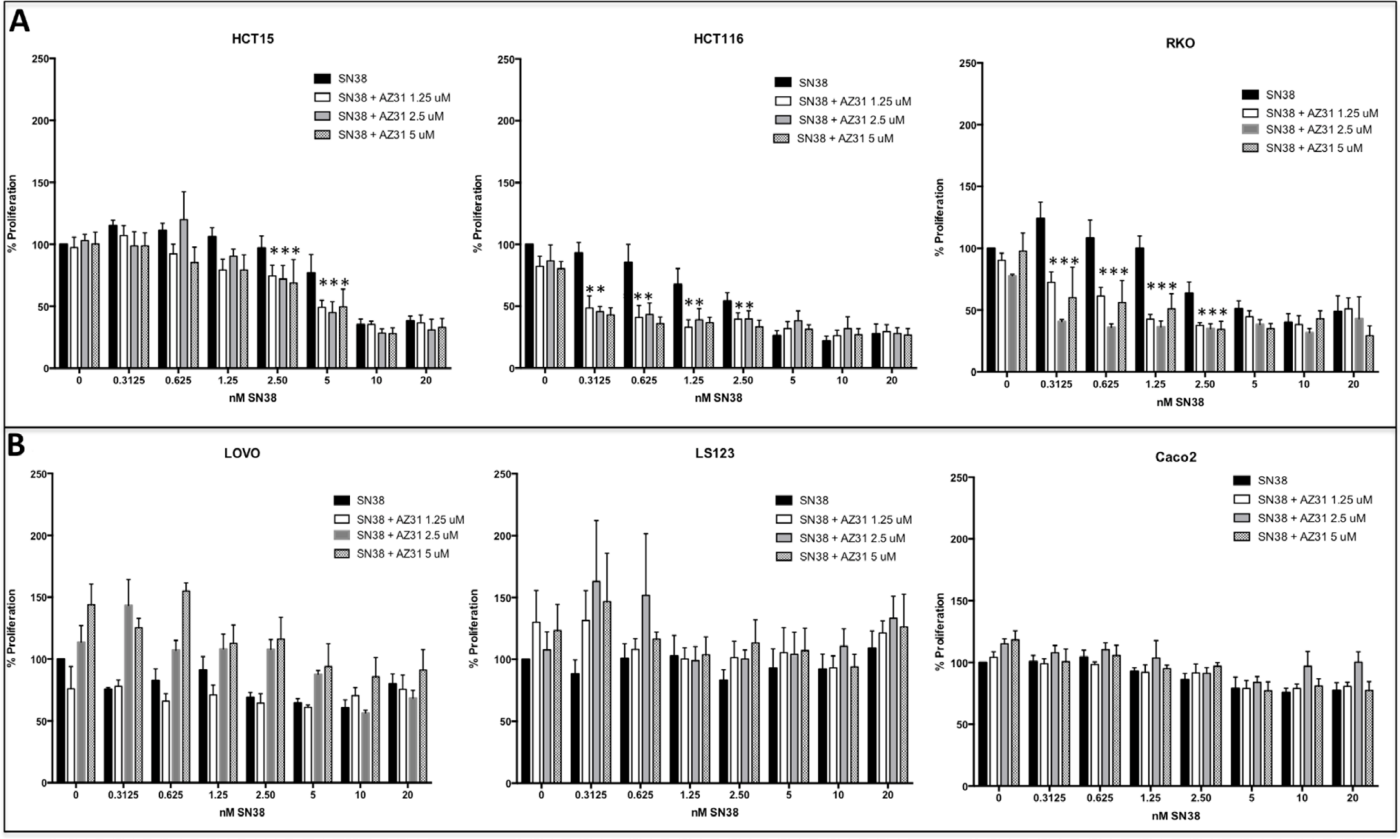
Antiproliferative effects of AZ31, SN38 and AZ31 + SN38 in CRC cell lines *in vitro* Six CRC cell lines were treated with AZ31 (dose 1.25, 2.5 or 5 μmol/L), SN38 (0.3125 – 20 nM) or AZ31 + SN38 and proliferation was determined by an SRB assay. (**A**) The 3 CRC cell lines HCT15, HCT116 and RKO all exhibited combinational sensitivity to AZ31 + SN38. (**B**) A combination effect was not observed in the CRC cell lines LOVO, LS123 and Caco2.

It has been previously shown that ATM inhibitors have little to no single agent antiproliferative effects on CRC cell lines, and the activity can be enhanced with the addition of the topoisomerase I inhibitor, SN38 [4]. Therefore, we evaluated the antiproliferative effects of AZ31 in combination with SN38 in CRC cell lines using a SRB assay. Cells were exposed for 72 hours to varying doses of each compound alone and in all possible combinations. Three CRC cell lines, HCT15, HCT116, and RKO demonstrated an enhanced combination effect (combination sensitive), when compared to the single agents at the different concentrations (Figure 1A). While the HCT15 cell line demonstrated enhanced combination effects at higher doses of SN38, the RKO and HCT116 were shown to be combination sensitive at the lower doses of SN38. In contrast, there were no significant combination treatment effects in CaCo2, LS123 and LOVO CRC cell lines (Figure 1B).

### Effects of AZ31 and SN38 on cell cycle arrest

**Figure 2:**
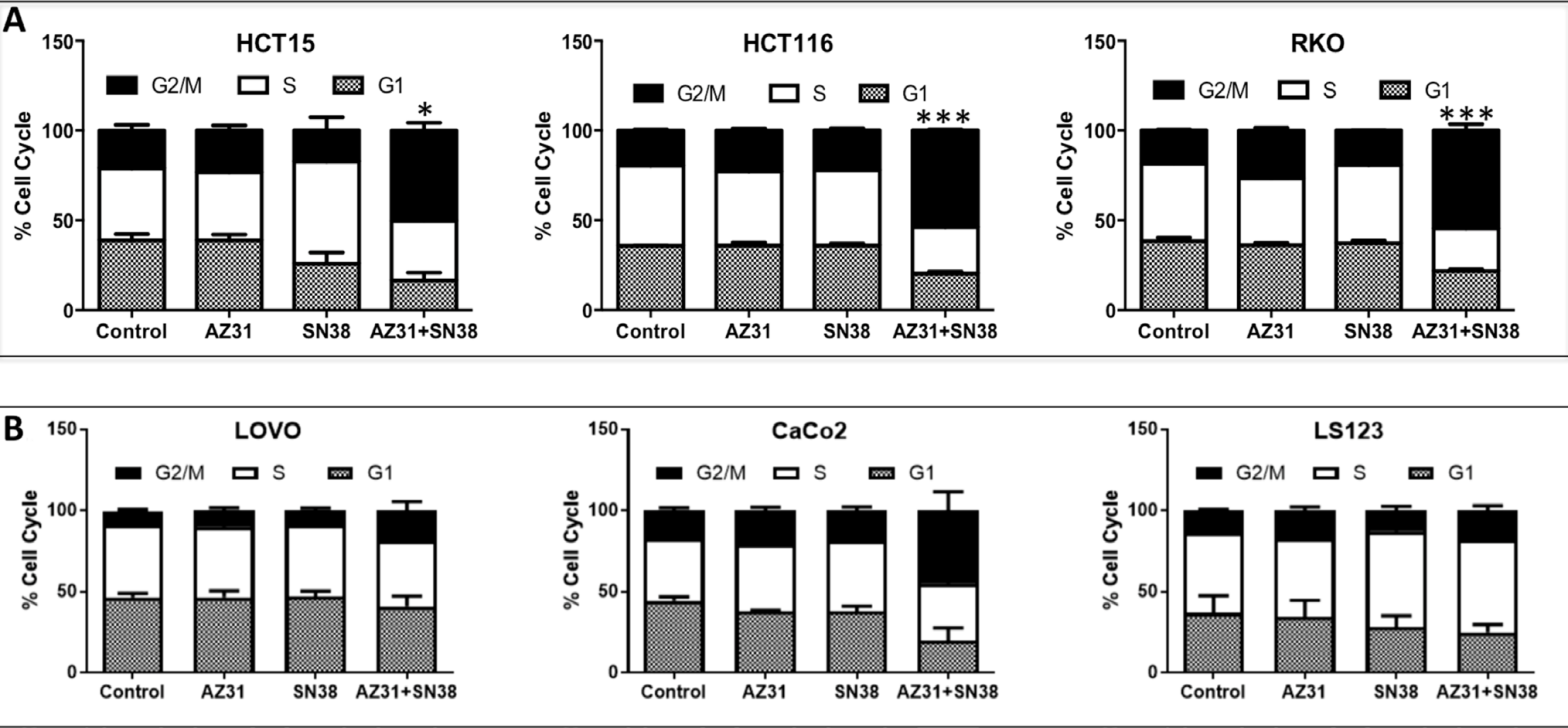
Cell cycle analysis of combination sensitive and resistant CRC cell lines (**A**) A significant increase in G2/M was seen in the CRC combination sensitive cell lines HCT15, HCT116 and RKO treated with AZ31 (1.25 uM) + SN38 (1.25 nM). (**B**) An increase in G2/M was only seen in the combination resistant CRC cell line CaCo2 treated with AZ31 (1.25 uM) + SN38 (1.25 nM) but not in LOVO and LS123.

It was initially thought that ATM inhibition would enhance the activity of DNA damaging agents by inducing apoptosis, however we did not observe any induction of apoptosis with treatment in combination sensitive and resistant CRC cell lines (Supplementary Figure 1). To determine the mechanism of enhanced anti-proliferative effects, we performed cell cycle analysis following treatment (AZ31, SN38 and AZ31 + SN38) for 24 hours. As shown in Figure 2A, all three of the combination sensitive CRC cell lines demonstrated a significant increase in G2/M arrest with AZ31 + irinotecan treatment when compared to control and single agent treatment. While an elevation in G2/M was seen in the CaCo2 CRC cell line, a G2/M increase was not evident in the LS123 and LOVO CRC cell lines (Figure 2B). All three of these CRC cell lines did not exhibit an anti-proliferative effect with combination treatment.

### Evaluation of the treatment effects on the ATM pathway

**Figure 3:**
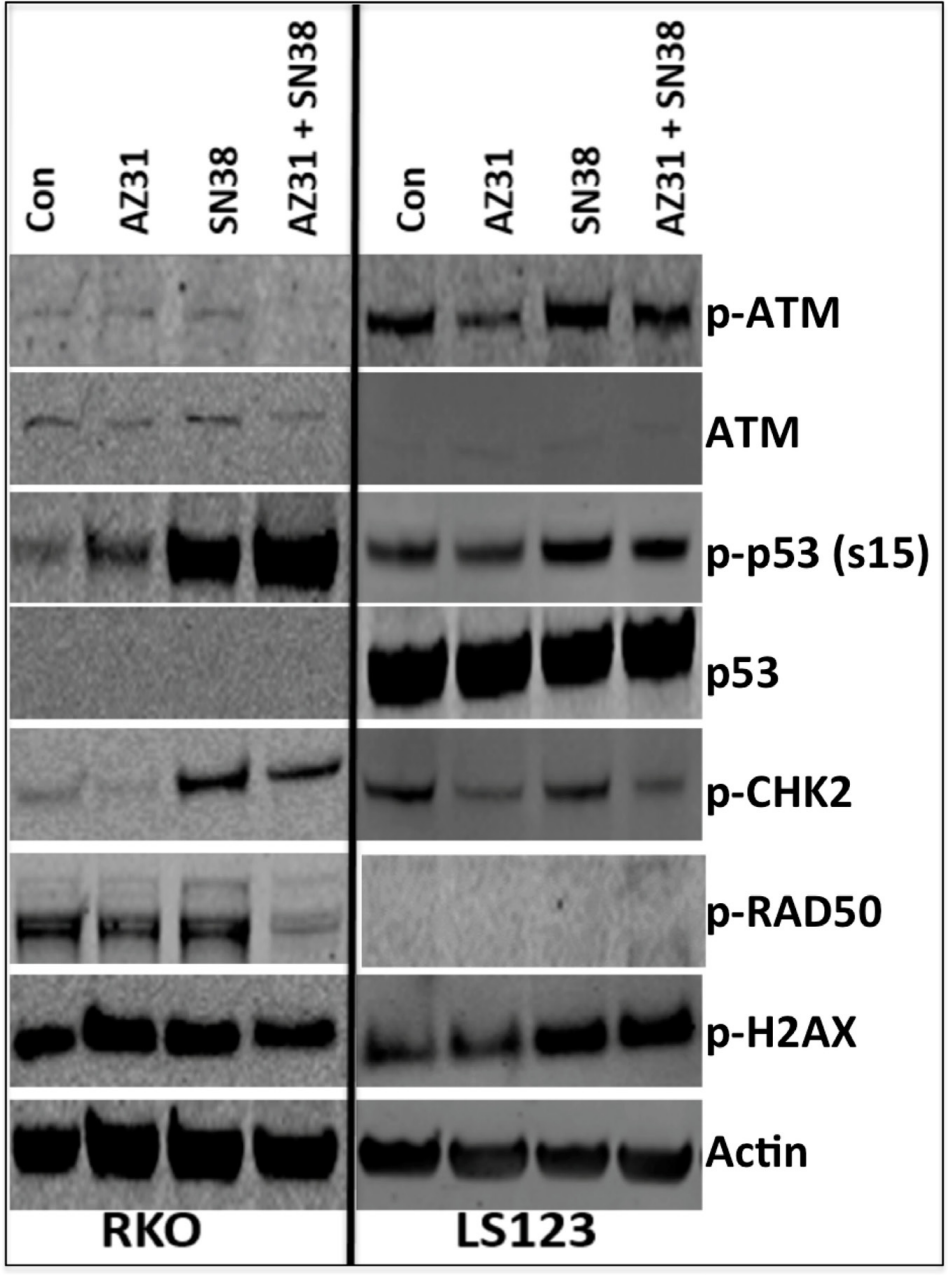
Immunoblot analysis of components of the ATM pathway RKO (combination sensitive) and LS123 (combination resistant) exhibited a reduction in the activation of ATM and CHK2. An increase in serine 15 p53 was observed only in the combination sensitive CRC cell line RKO. A decrease RAD50 activation was only seen in the RKO cell line treated with AZ31 (1.25 uM) + SN38 (1.25 nM).

To further understand the mechanism whereby SN38 in combination with ATM inhibition facilitates anti-proliferative effects and G2/M cell cycle arrest, we examined treatment effects on the ATM pathway by immunoblotting in the combination sensitive (RKO) and combination resistant (LS123) CRC cell lines. Overall, in both CRC cell lines, we observed a reduction in the activation of ATM and CHK2 with AZ31 + SN38 treatment when compared to SN38 alone (Figure 3). In addition, activation of p53 was markedly increased following exposure to SN38 as well as with AZ31 + SN38 in the RKO combination sensitive cell line. This was not the case in the LS123 cell line where p-p53 was only slightly increased following SN38 and was slightly reduced with combination treatment. Of note, no total p53 was observed in the RKO cell line. Evaluation of p-RAD50, a protein that plays an important role in cell cycle checkpoint signaling and double-stranded break repair was decreased with combination treatment. Interestingly, activation of RAD50 was not seen in any groups in the LS123 combination resistant CRC cell line.

### Investigation of ATM inhibition in combination with Irinotecan in CRC patient derived xenograft models

**Figure 4:**
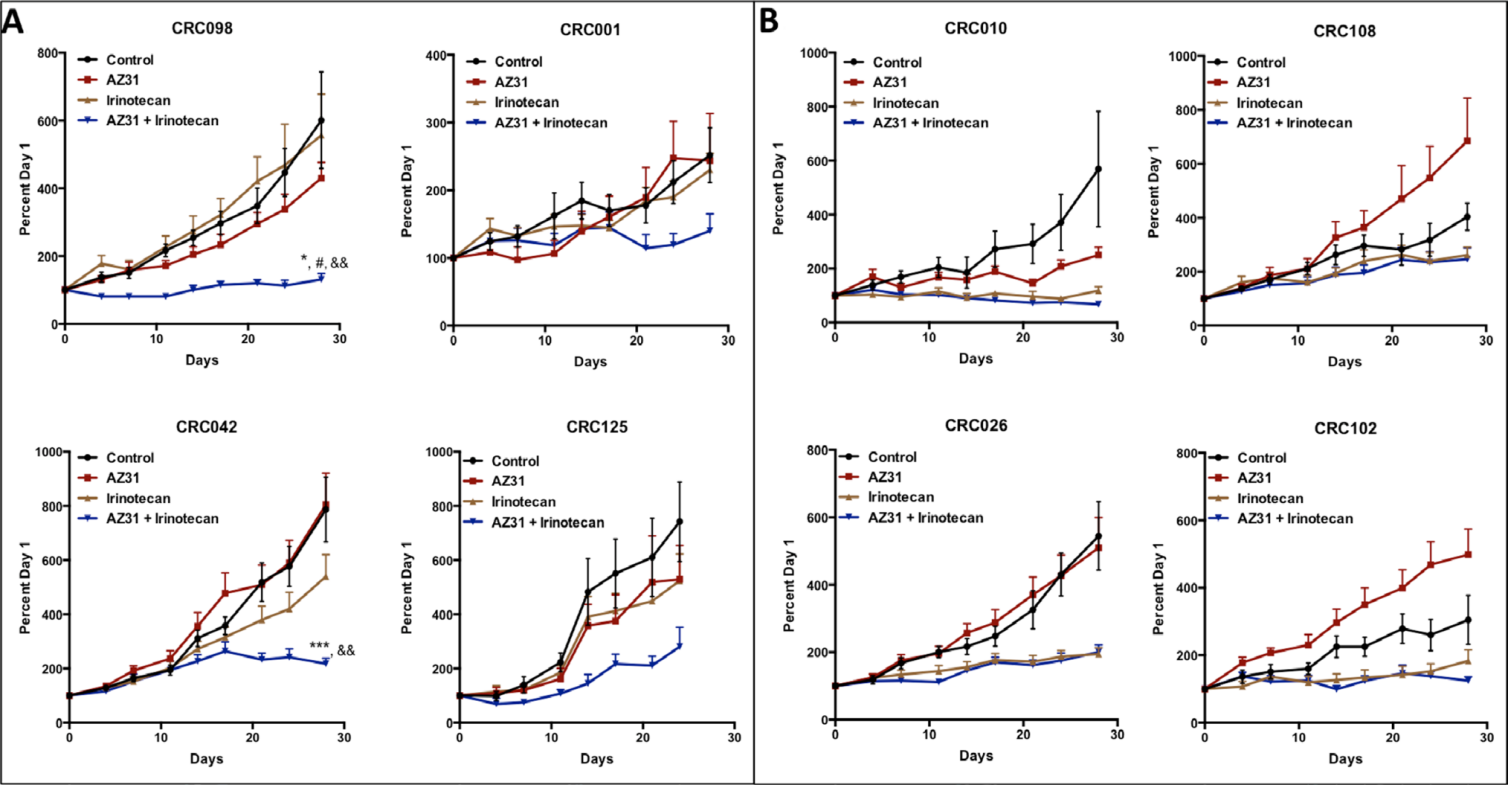
The effects of treatment on tumor growth in CRC PDX models Eight unique CRC PDX models were treated (vehicle, AZ31, irinotecan, or AZ31 + irinotecan) for 28 days. Irinotecan was dosed on day 1 followed by AZ31 on day 2, 3 and 4 of each week for 4 weeks. (**A**) CRC001, CRC042, CRC098 and CRC125 all exhibited a decrease in tumor growth in response to combination when compared to vehicle, AZ31 and irinotecan. (**B**) No combinational differences were observed in CRC010, CRC026, CRC102 and CRC108 when compared to single agent irinotecan. Each data point represents an average of ≥10 tumors per treatment group. Data presented as mean ± SEM, ANOVA (Dunnett’s) p values: ^*^p < 0.05, ^***^p < 0.001, vs. control; ^#^*p* < 0.05, vs. AZ31; ^&&^*p* < 0.01, vs. IRN.

To determine the *in vivo* efficacy of the novel ATM inhibitor (AZ31), we assessed treatment effects on tumor growth of eight unique CRC PDX models. AZ31 is a potent and selective ATM inhibitor with good oral exposure in preclinical species (Degorce *et al*, 2016). Supplementary Table 2 shows the patient characteristics and mutational status of KRAS, PIK3CA, BRAF, TP53, ATM and NRAS of these eight tumors. All eight tumors studied were ATM wild type. A combination treatment effect was observed in 4 out of 8 (50%) CRC explants (CRC001, 042, 098, 125) (Figure 4A). Interestingly, all of these PDX models exhibited resistance to irinotecan monotherapy. In contrast, no combination treatment effect was observed in the PDX models (CRC010, CRC108, CRC026, and CRC102) that displayed sensitivity to irinotecan treatment (Figure 4B). Furthermore, comparison of tumor growth inhibitor index at end of study between the four combination sensitive (resistant to irinotecan) vs. four combination resistant (sensitive to irinotecan) PDX models revealed a significant difference between the two groups with respect to irinotecan treatment (Supplementary Figure 2).

### Pharmacokinetic and pharmacodynamic analysis of AZ31 and/or irinotecan in the combination sensitive and resistant PDX models

**Figure 5:**
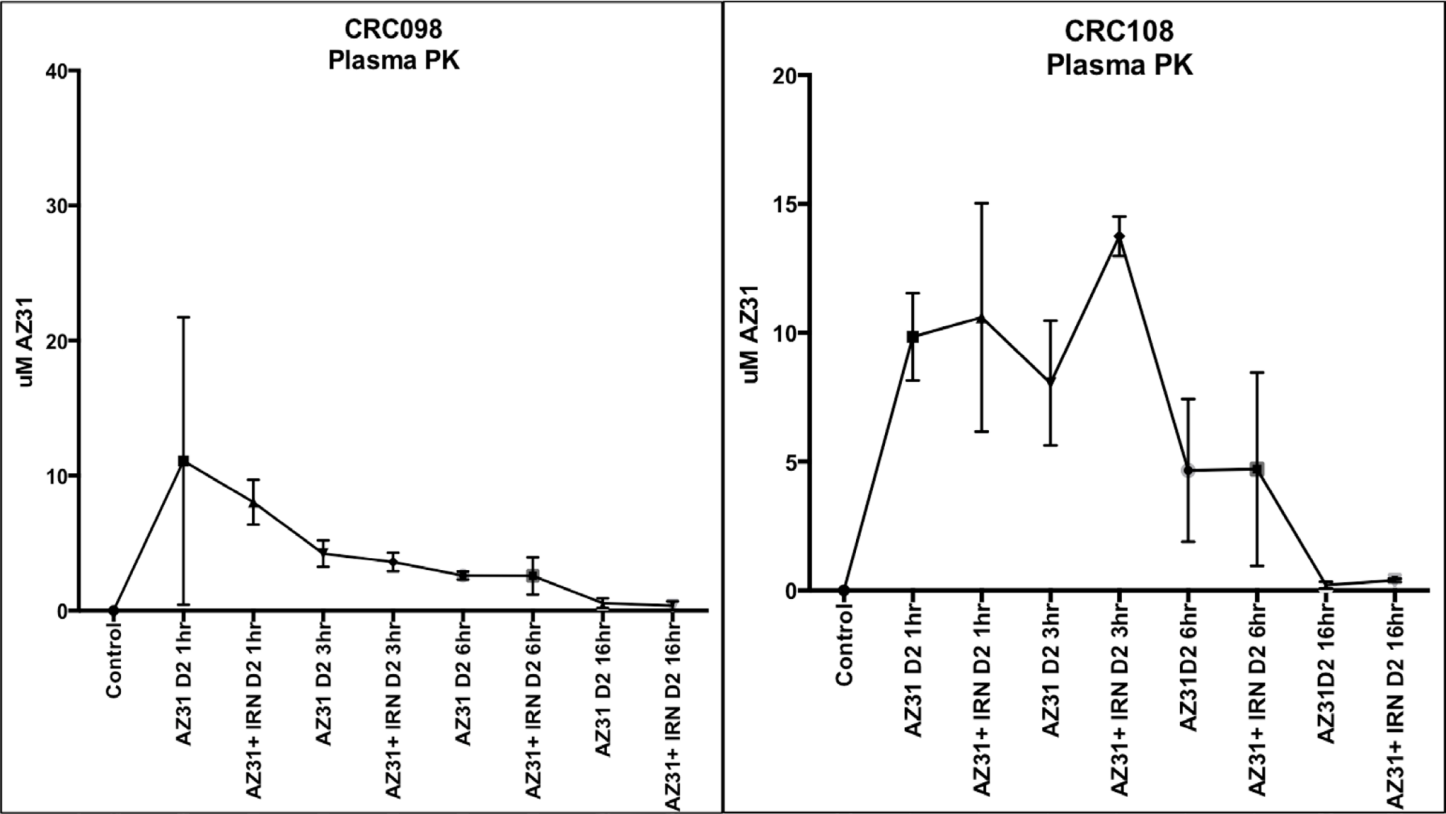
Pharmacokinetic evaluation of plasma concentrations of AZ31 in CRC098 (combination sensitive) and CRC108 (combination resistant) (**A**) Minimal concentrations were detected in CRC098 and 108 16 hours after AZ31 administration in the AZ31 and AZ31 + irinotecan treated groups.

To ensure that the differences observed in the combination sensitive and resistant models were not due to differing plasma levels of AZ31 and to confirm that irinotecan does not interfere with drug levels, pharmacokinetic analysis was performed. Mice were dosed for indicated times and blood was obtained from at least three individual mice and plasma drug concentrations were determined. As displayed in Figure 5, there were no differences in plasma levels between CRC098 (combination sensitive) and CRC108 (combination resistant) following dosing of AZ31 and AZ31 + irinotecan.

**Figure 6:**
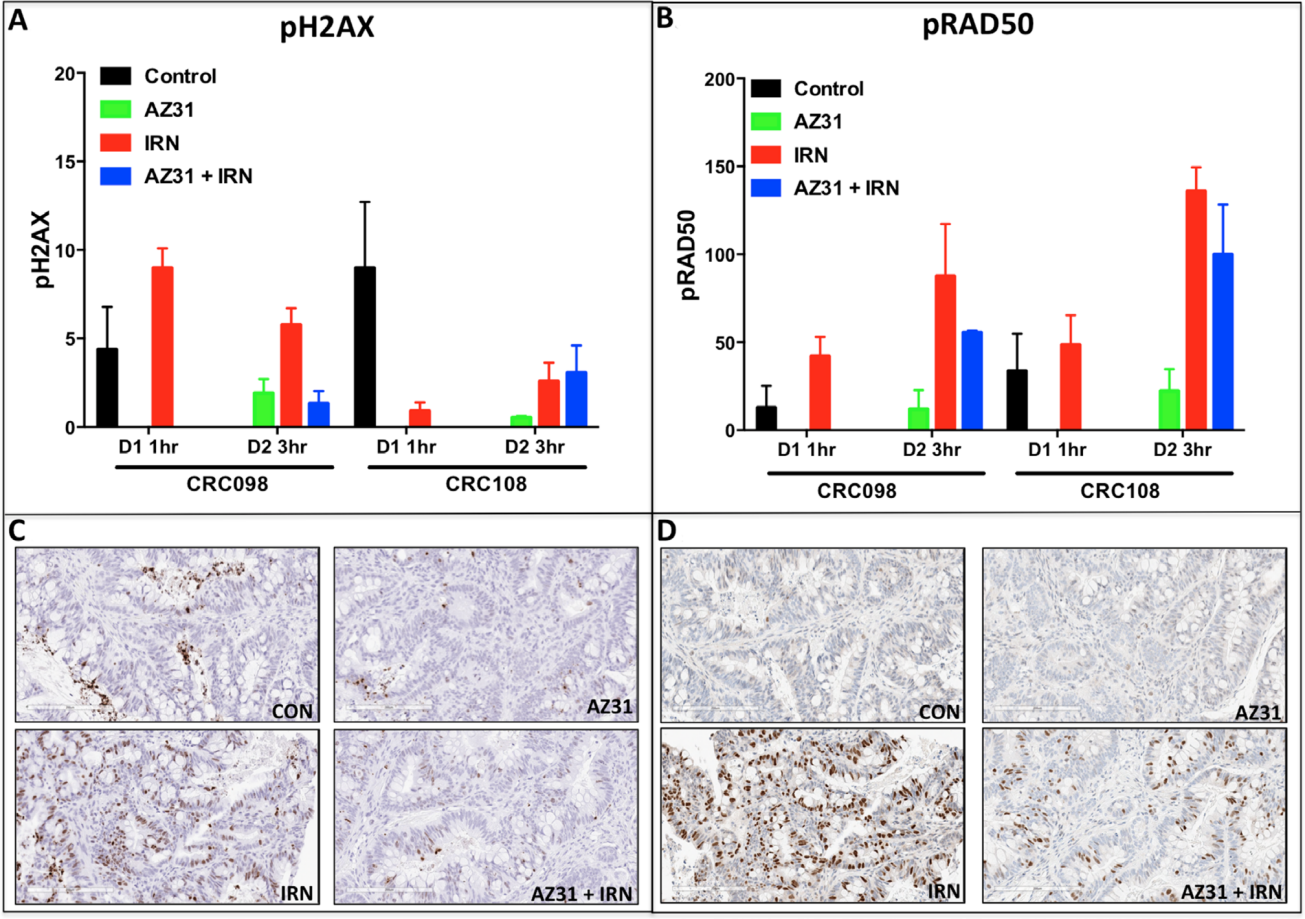
Immunohistochemistry analysis of p-H2AX and p-RAD50 in CRC098 (combination sensitive) and CRC108 (combination resistant) (**A**–**B**) A marked increase in the activation of H2AX and RAD50 was demonstrated on day 2– 3 hr after dosing in both CRC098 and CRC108. A reduction in the phosphorylation of H2AX and RAD50 in response to combination treatment was evident only in the combination sensitive CRC098. Of note, only irinotecan was administered on day 1 for CRC098 and CRC108 in order to assess irinotecan effects at 1 hour on the activation of the pH2AX and p-RAD50. (**C**–**D**) Representative depictions of p-H2AX and pRAD50 are shown.

Next, we investigated the pharmacodynamics effects of AZ31 and/or irinotecan on the activation of the ATM downstream targets H2AX and RAD50 by immunohistochemistry. As shown in Figure 6A and 6B, in CRC098 and CRC108 there was a striking increase in the activation of H2AX and RAD50 3 hours after dosing; however, only in the CRC098 model, the addition of the ATM inhibitor (AZ31) with irinotecan reduced the phosphorylation of H2AX and RAD50 (Figure 6A–6D). Representative photographs of p-H2AX and p-RAD50 immunostaining in CRC098 are displayed in Figure 6C and 6D. A combinational decrease was not observed in the CRC108 model with respect to the activation of H2AX and RAD50 (Figure 6A–6B).

## DISCUSSION

The ATM signaling cascade is an integral component in the regulation of cell cycle checkpoints and repair of DNA double-stranded breaks (DSB) [12, 13]. Cells deficient in ATM have an altered response to DNA damage, resulting in a propensity of DNA damaging events. Given the importance of ATM as a master regulator in the cell ensuring DNA replication fidelity, it makes ATM an attractive target to enhance the sensitivity of tumor cells to DNA damaging chemotherapeutic agents and radiation.

Commercially, there are only a few ATM inhibitors available, which include KU59403 and KU60019. These drugs have shown some activity in colorectal carcinoma, breast cancer and glioma but are not currently under clinical investigation [4, 15–17]. In particular, KU59403 significantly enhanced the cytotoxic effects of etoposide, campthothecin and doxorubicin in CRC and breast cancer cell lines [4]. In our preclinical study evaluating the treatment effects of the selective and novel ATM inhibitor (AZ31) in combination with SN38, we demonstrated synergistic combination effects *in vitro* in 3 out of 6 CRC cells. To further investigate the anti-proliferative mechanisms in combination sensitive and resistant cell lines, we assessed the effects of AZ31 + SN38 on apoptosis and cell cycle. While an induction in apoptosis was not evident with combination treatment, a significant increase in G2/M cell cycle arrest was seen in the combination sensitive cell lines. This finding coincides with another study whereby ATM inhibition resulted in G2/M arrest [15]. These observed results suggest that the antitumor effects of AZ31 + SN38 are cytostatic rather than cytotoxic.

Next, we utilized our CRC PDX models and tested the effects of AZ31 as a single agent and in combination with irinotecan. Irinotecan was dosed on day 1 followed by AZ31 on day 2, 3 and 4 of each week for 4 weeks. Similar to the results seen *in vitro*, combinational treatment effects were observed in 4 out of 8 CRC PDX models tested. Interestingly, the models that exhibited a combination treatment effect were the CRC PDX models that displayed resistance to irinotecan monotherapy, suggesting a potential role for ATM activation as a mechanism of irinotecan resistance in this subset of tumors. In contrast, in the PDX models where there were no combinational treatment effects, the antitumor activity was mainly driven by irinotecan. These results indicate that blocking the ATM pathway in combination with irinotecan may be an effective therapeutic strategy especially in patients that exhibit resistance to irinotecan therapy.

In addition to tumors exhibiting irinotecan resistance and combination sensitivity, we identified an association ( *p* = 0.02– 2 × 2 fisher exact test- data not shown) between PIK3CA mutation and combination sensitivity when we combined CRC cell lines and PDX models. The HCT15, HCT116, RKO cell lines and CRC042, CRC098 PDX models all harbor a mutation in the *PIK3CA* gene [18]. Other studies have shown a relationship between high expression of PI3K [17] or PTEN-deficient tumor cells [15] with increased response to ATM pathway inhibition. Given these findings, additional studies are needed to delineate the underlying mechanism whereby PIK3CA mutant tumors are more susceptible to the combinational effects of ATM pathway inhibition and irinotecan. Furthermore, molecular profiling of combination sensitive and resistant tumors may yield a better understanding of alternative pathway differences between these groups. Finally, no association was seen with respect to *TP53* mutational status and response to combinational treatment although other studies indicated that *TP53* mutational status could be a marker of response [4, 19].

Pharmacokinetic investigation of AZ31 as a single agent and in combination with irinotecan revealed that plasma concentrations of AZ31 were highest 1-hour after administration followed by a stepwise decrease at 3, 6 and 16 hour in the combination sensitive CRC098. Similar findings were observed in the combination resistant CRC108, plasma concentrations were highest at 1 hour but decreased at 6 and 16hr following AZ31 administration. These results indicate that plasma concentrations are similar in both models and this is not the reason for the lack of response in CRC108.

Given the importance of ATM in activating many different downstream targets that are involved in cell cycle arrest and DNA repair, we set out to investigate the treatment effects on the activation of RAD50, CHK2, p53 and H2AX. We showed that the activation of p53 (serine 15) was more pronounced after exposure to SN38 and AZ31 + SN38 in the CRC cell line that was combination sensitive compared with the CRC cell line that did not display a combination effect. ATM-dependent activation of p53 plays a fundamental role in regulating genes that are involved in cell cycle checkpoint and apoptosis [11]. Also, treatment with AZ31 + SN38 resulted in a decrease in the activation of CHK2 and RAD50, which play an important role in cell cycle regulation and DNA repair respectively [11]. In our PDX tumors, both H2AX and RAD50 were strongly activated after irinotecan treatment and decreased activation was only observed in the combination sensitive CRC098 with AZ31 + irinotecan. These results suggest that inhibition of ATM alters the downstream response to irinotecan DNA damaging effects resulting in cell cycle arrest and disruption of DNA repair.

The results of this preclinical study implicate the ATM pathway as an important facilitator of resistance to irinotecan monotherapy in a subset PDX models. These findings support further investigation of this combinational therapy for the treatment of CRC patients. Certainly, the identification of a predictive biomarker of irinotecan resistance or combinational response will further aid in the clinical development of this combinational therapy.

## MATERIALS AND METHODS

### Chemicals and reagents

AZ31 was provided by AstraZeneca (Waltham, MA). For *in vitro* work, AZ31 was dissolved in 100% DMSO at a concentration of 10 mmol/L. For *in vivo* studies, AZ31 was formulated in 10% v/v DMSO + 90% v/v Captisol at 30% w/v and dosed as described below.

### Cell lines, culture, proliferation and apoptosis

The CRC cell lines (HCT15, HCT116, RKO, LOVO, LS123, Caco2) were obtained from ATCC (Manassas, VA) and routinely cultured in RPMI 1640. All medium was supplemented with 10% FBS, 1% penicillin–streptomycin, and 1% MEM nonessential amino acids. All cells were kept at 37°C under an atmosphere containing 5% CO_2_. All CRC cell lines used in this study have been fully characterized and authenticated in the University of Colorado Cancer Center DNA Sequencing and Analysis Core. Cytotoxic effects on the cell lines were determined using the sulforhodamine B assay [20]. Briefly, cells in logarithmic growth phase were transferred to 96-well flat-bottomed plates with lids. Cell suspensions (100 μL) containing 1500 to 3000 viable cells were plated into each well and incubated overnight before exposure to varying concentrations of AZ31 as a single agent or in combination for 72 hours. After drug treatment, media was removed and cells were fixed with cold 10% TCA for 30 minutes at 4°C. Cells were then washed with water and stained with 0.4% sulforhodamine B for 30 minutes at room temperature. The plates were washed with 1% acetic acid followed by stain solubilization with 10 mM Tris. The plate was then read on a plate reader (Biotek Synergy 2) set at an absorbance wavelength of 565 nm. For apoptosis determination, cells were plated as described above in 96-well, white-walled plates and allowed to adhere overnight. Cells were then exposed to indicated concentrations for 6, 12, and 24 hours. Apoptosis was determined using the Caspase Glo 3/7 (Promega, Milwaukee WI) following the manufactures’ instructions.

### Cell cycle analysis

Cells (1 × 10^5^ per well) were added in a 6-well plate containing 2 mls of complete media and allowed to adhere overnight. The next day, the cells were treated with AZ31, SN38 or AZ31 + SN38 at the indicated concentrations. After 24 hours of treatment the cells were washed 2 times with 1xPBS and then resuspended in Krishan’s stain. The cells were stained overnight in the refrigerator and analyzed by flow cytometry at the University of Colorado Cancer Center Flow Cytometry Core Facility. Each experiment was repeated 3 times.

### Patient-derived xenograft studies

Patient-derived colorectal adenocarcinoma tumor specimens (CRC001, CRC010, CRC026, CRC042, CRC098, CRC102, CRC108, and CRC125) were obtained from consenting patients at the University of Colorado Hospital in accordance with a protocol approved by the Colorado Multiple Institutional Review Board (08-0439). Four-to-six week-old female athymic nude mice were obtained from Harlan laboratories (Indianapolis, IN) under an approved research protocol by the Institutional Animal Care and Use Committee. The tumor pieces were implanted in mice and expansion of the F1-F3 generations was carried out as previously described [21, 22]. Tumors were expanded in both the left and right flanks of 5–6 mice (10 evaluable tumors per group). Mice were randomized into vehicle, AZ31, irinotecan or AZ31 + irinotecan groups when tumor volumes reached ∼200 mm^3^. Mice were treated daily with AZ31 (100 mg/kg – daily × 3) by oral gavage or irinotecan (15 mg/kg – weekly) by ip for at least 28 days. Mice treated with combination therapy were treated with irinotecan on day 1 followed by AZ31 daily for three consecutive days. Mice were monitored daily for signs of toxicity and tumor size was evaluated twice per week by caliper measurements using the following formula: tumor volume = [length × width^2^]* 0.52.

### Immunoblotting

Cells were seeded in 6-well plates and allowed to attach for 24 h. Cells were then incubated for 24 hours with indicated concentrations of AZ31 and/or SN38. Cells were then washed with PBS and lysed with RIPA buffer (Cell Signaling, Danvers, MA). After sonication and centrifugation, a total of 30 μg of protein lysate was loaded onto a NuPage gel (Life Technologies, Carlsbad, CA), electrophoresed, and transferred to a nitrocellulose membrane using the Pierce G2 FastBlotter (Thermo Fisher, Rockford, IL). The membrane was blocked and probed overnight with primary antibodies (Cell Signaling Technologies (Danvers, MA) at a concentration 1:1000: p-ATM (catalog# 13050), ATM (catalog# 2873), p-p53 (catalog# 9284), p53 (catalog# 2527), p-CHK2 (catalog# 2665), p-RAD50 (catalog# 14223), p-H2AX (catalog# 9718) and actin (catalog# 4970). The next day the membranes were washed for 10 minutes 3X with TBS/Tween 20, and then probed with DyLight secondary antibodies 1:15,000 (Cell signaling, Danvers, MA), and imaged using the Licor Odyssey (Licor, Lincoln, NE).

### Plasma bioanalysis

Plasma was obtained from CRC-098 and CRC-108 models treated with vehicle, AZ31, irinotecan or irinotecan + AZ31. Each plasma sample (25 μl) was prepared using an appropriate dilution factor, and compared against an 11-point standard calibration curve (1-10000 nM) prepared in DMSO and spiked into blank plasma. Acetonitrile (100 μl) was added with the internal standard, followed by centrifugation at 3000 rpm for 10 minutes. Supernatant (50 μl) was then diluted in 300 μl water and analyzed via UPLC-MS/MS (Supplementary Table 1). For each sample, there were 3 mice used for the analysis.

### Immunohistochemistry (IHC)

Four mm sections were taken from Tissue Microarrays (TMA) containing formalin fixed and paraffin embedded (FFPE) tissues from CRC-098 and CRC-108 models treated with vehicle, AZ31, irinotecan or irinotecan + AZ31 (*n* = 3 cores per treatment time point, per model).

For phosphorylated H2AX IHC staining, tissues were dewaxed in xylene, rehydrated in graded alcohols and water and antigen retrieved at 110°C pH 9 retrieval buffer for 2 min (Dako). The LabVision autostainer (Thermo Scientific) was used for staining; 10 min 3% hydrogen peroxide, 20 min serum free protein block (Dako), 60 min phospho-histone H2A.X (Ser139) (p-H2AX) 20E3 rabbit monoclonal antibody (CST) at 0.67 µg/ml in TBS-Tween (0.05%), 30 min EnVision™ + System-HRP labelled polymer (Rabbit) (Dako), and 10 min diaminobenzidine (DAB) (Dako). Washes were done with TBS-Tween (0.05%). Carazzi’s haematoxylin was used to counterstain the nuclei.

For pRAD50 staining, tissue sections were stained using the Ventana Discovery Ultra (Roche). A 24 min EZ prep deparaffinization step at 69°C was performed followed by a 32 min CC1 antigen retrieval at 98°C and a 32 min block with antibody diluent with casein (Roche) at 36°C. Phospho-Rad50 (Ser635) (pRAD50) rabbit polyclonal antibody (CST) at 1 µg/ml in PSS antibody diluent was added to the sample at 32 min 36°C (Roche) followed by a 8 min 36°C anti-rabbit HQ (Roche), 8 min 36°C anti-HQ HRP (Roche) and DAB staining (Discovery Chromomap DAB kit). Washes were performed with reaction buffer (Roche). Hematoxylin II and bluing reagent (Roche) was used for nuclei counterstain.

### Image analysis

The Aperio AT2 scanner (Leica) was used to scan the IHC stained slides. Classifiers and image analysis algorithms were developed for each biomarker using HALO image analysis (Indica Labs) and used to analyse positive staining on the sildes. For p-H2AX staining, percentage of positive staining pixels in tumour regions were analysed using an area quantification algorithm and tumor tissue classifier. For pRAD50 staining, a cytonuclear algorithm and tumour tissue classifier was used to quantify the percentage of nuclei with strong (3+), moderate (2+), weak (1+) or negative pRAD50 staining in tumour regions. H-Score was calculated as: [(% 1+ cells) + (% 2+ cells *2) + (% 3+ cells *3)].

### Statistical analysis

For combinational treatment, a one-way analysis of variance (ANOVA) was used to determine whether the means were significantly different overall at end of treatment in the PDX model, proliferation and cell cycle analysis. If the overall means were significantly different, we carried out a pair-wise comparison. *P* values were adjusted using Dunnett’s test for multiple comparisons. SE of the mean was indicated for each value by a bar. All analyses were carried out using Graph-Pad Prism version 5.0c for Windows (GraphPad Software, San Diego).

## SUPPLEMENTARY MATERIALS FIGURES AND TABLES



## References

[1] Siegel RL, Miller KD, Jemal A (2016). Cancer statistics, 2016. CA Cancer J Clin.

[2] Gustavsson B, Carlsson G, Machover D, Petrelli N, Roth A, Schmoll HJ, Tveit KM, Gibson F (2015). A review of the evolution of systemic chemotherapy in the management of colorectal cancer. Clin Colorectal Cancer.

[3] Berkovich E, Monnat RJ, Kastan MB (2007). Roles of ATM and NBS1 in chromatin structure modulation and DNA double-strand break repair. Nat Cell Biol.

[4] Batey MA, Zhao Y, Kyle S, Richardson C, Slade A, Martin NM, Lau A, Newell DR, Curtin NJ (2013). Preclinical evaluation of a novel ATM inhibitor, KU59403, *in vitro* and *in vivo* in p53 functional and dysfunctional models of human cancer. Mol Cancer Ther.

[5] Cremona CA, Behrens A (2014). ATM signalling and cancer. Oncogene.

[6] Yim KL, Cunningham D (2009). Chemotherapy: Optimizing irinotecan regimens for colorectal cancer. Nat Rev Clin Oncol.

[7] Cunningham D, Maroun J, Vanhoefer U, Van Cutsem E (2001). Optimizing the use of irinotecan in colorectal cancer. Oncologist.

[8] Goodarzi AA, Noon AT, Deckbar D, Ziv Y, Shiloh Y, Lobrich M, Jeggo PA (2008). ATM signaling facilitates repair of DNA double-strand breaks associated with heterochromatin. Mol Cell.

[9] Hirao A, Cheung A, Duncan G, Girard PM, Elia AJ, Wakeham A, Okada H, Sarkissian T, Wong JA, Sakai T, De Stanchina E, Bristow RG, Suda T (2002). Chk2 is a tumor suppressor that regulates apoptosis in both an ataxia telangiectasia mutated (ATM)-dependent and an ATM-independent manner. Mol Cell Biol.

[10] Huen MS, Chen J (2008). The DNA damage response pathways: at the crossroad of protein modifications. Cell Res.

[11] Shiloh Y, Ziv Y (2013). The ATM protein kinase: regulating the cellular response to genotoxic stress, and more. Nat Rev Mol Cell Biol.

[12] Jekimovs C, Bolderson E, Suraweera A, Adams M, O’Byrne KJ, Richard DJ (2014). Chemotherapeutic compounds targeting the DNA double-strand break repair pathways: the good, the bad, and the promising. Front Oncol.

[13] Lavin MF (2008). Ataxia-telangiectasia: from a rare disorder to a paradigm for cell signalling and cancer. Nat Rev Mol Cell Biol.

[14] Pitts TM, Davis SL, Eckhardt SG, Bradshaw-Pierce EL (2014). Targeting nuclear kinases in cancer: development of cell cycle kinase inhibitors. Pharmacol Ther.

[15] McCabe N, Hanna C, Walker SM, Gonda D, Li J, Wikstrom K, Savage KI, Butterworth KT, Chen C, Harkin DP, Prise KM, Kennedy RD (2015). Mechanistic Rationale to Target PTEN-Deficient Tumor Cells with Inhibitors of the DNA Damage Response Kinase ATM. Cancer Res.

[16] Zhu Y, Mao C, Wu J, Li S, Ma R, Cao H, Ji M, Jing C, Tang J (2014). Improved ataxia telangiectasia mutated kinase inhibitor KU60019 provides a promising treatment strategy for non-invasive breast cancer. Oncol Lett.

[17] Vecchio D, Daga A, Carra E, Marubbi D, Raso A, Mascelli S, Nozza P, Garrè ML, Pitto F, Ravetti JL, Vagge S, Corvò R, Profumo A (2015). Pharmacokinetics, pharmacodynamics and efficacy on pediatric tumors of the glioma radiosensitizer KU60019. Int J Cancer.

[18] Arcaroli JJ, Quackenbush KS, Powell RW, Pitts TM, Spreafico A, Varella-Garcia M, Bemis L, Tan AC, Reinemann JM, Touban BM, Dasari A, Eckhardt SG, Messersmith WA (2012). Common PIK3CA mutants and a novel 3’ UTR mutation are associated with increased sensitivity to saracatinib. Clin Cancer Res.

[19] Sullivan KD, Palaniappan VV, Espinosa JM (2015). ATM regulates cell fate choice upon p53 activation by modulating mitochondrial turnover and ROS levels. Cell Cycle.

[20] Waaler J, Machon O, von Kries JP, Wilson SR, Lundenes E, Wedlich D, Gradl D, Paulsen JE, Machonova O, Dembinski JL, Dinh H, Krauss S (2011). Novel Synthetic Antagonists of Canonical Wnt Signaling Inhibit Colorectal Cancer Cell Growth. Cancer Res.

[21] Rubio-Viqueira B, Jimeno A, Cusatis G, Zhang X, Iacobuzio-Donahue C, Karikari C, Shi C, Danenberg K, Danenberg PV, Kuramochi H, Tanaka K, Singh S, Salimi-Moosavi H (2006). An *in vivo* platform for translational drug development in pancreatic cancer. Clin Cancer Res.

[22] Dangles-Marie V, Pocard M, Richon S, Weiswald LB, Assayag F, Saulnier P, Judde JG, Janneau JL, Auger N, Validire P, Dutrillaux B, Praz F, Bellet D, Poupon MF (2007). Establishment of human colon cancer cell lines from fresh tumors versus xenografts: comparison of success rate and cell line features. Cancer Res.

