# Supplementary material for: The novel ATM inhibitor (AZ31) enhances antitumor activity in patient derived xenografts that are resistant to irinotecan monotherapy
Source: Oncotarget. 2017 Dec 5;8(67):110904–13. doi: 10.18632/oncotarget.22920 (PMC5762293; doi:10.18632/oncotarget.22920)
Supplement: Supplementary file 1 [file oncotarget-08-110904-s001.pdf]

The novel ATM inhibitor (AZ31) enhances antitumor activity in patient derived xenografts that are resistant to irinotecan monotherapy

SUPPLEMENTARY MATERIALS

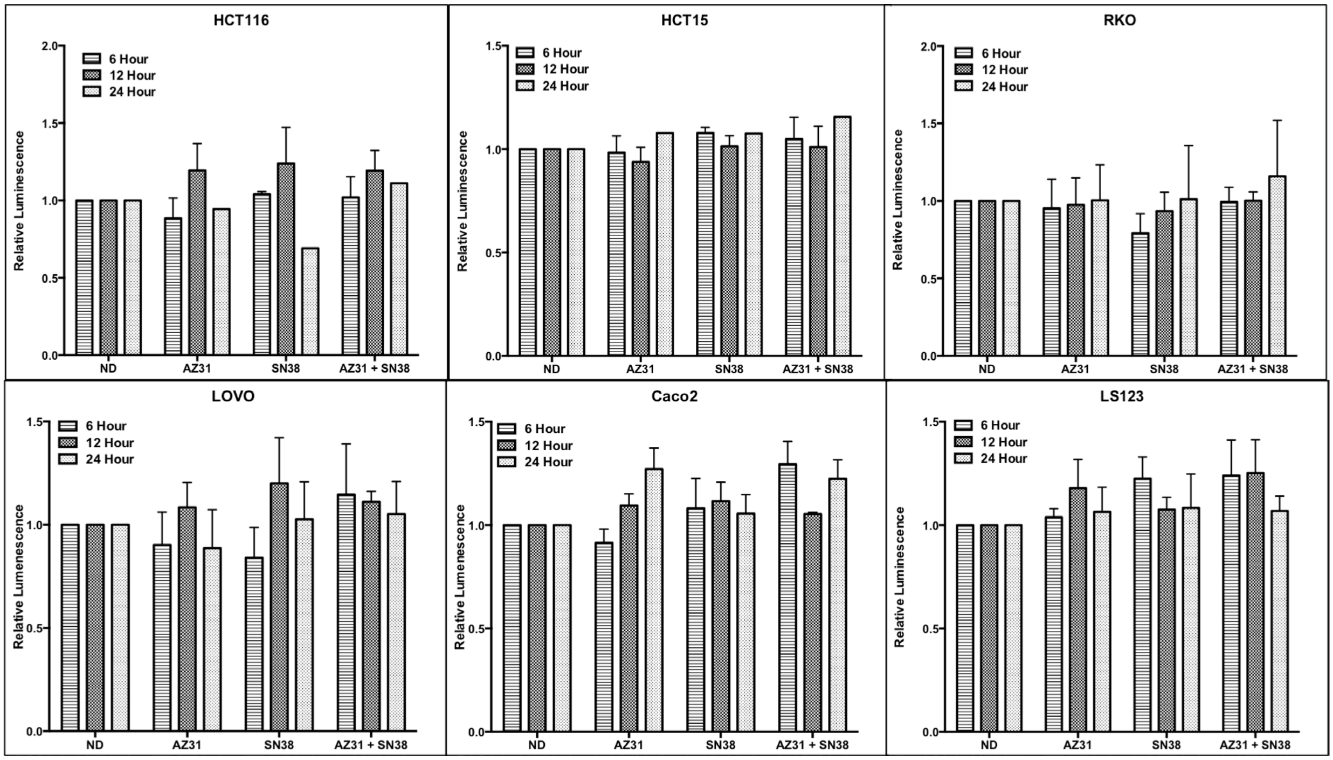

Supplementary Figure 1: Evaluation of treatment effects on apoptosis on combination sensitive and resistant CRC cell lines.

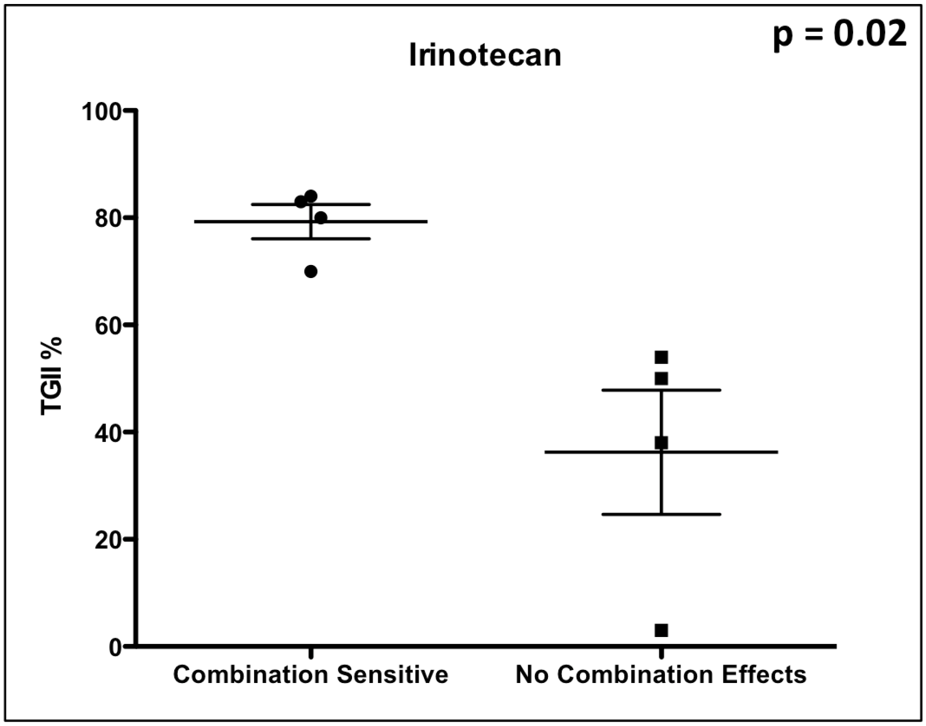

Supplementary Figure 2: Comparsion of irinotecan monotherapy between CRC PDX models sensitive (CRC098, 001, 042 and 125) and PDX models without a combination effect (CRC010, 108, 026 and 102) to AZ31 + Irinotecan. A TGII, a standardized measure of tumor growth, was calculated for each CRC explant using the following formula: TGII = (tumor volume of TX on Day 28 – tumor volume of TX on Day 0)/(tumor volume of Con on Day 28 – tumor volume of Con on Day 0) × 100. Plotted is the %TGII for each PDX model in response to irinotecan.

Supplementary Table 1: Method to determine plasma drug concentrations of AZ31

**Mass Spec**	Waters Xevo TQS		
**UPLC system**	Waters Acquity i-Class		
**Column**	Phenomenex Kinetix C18 50 × 2.1, 1.7u		
**Solvent A**	95% Water, 5% MeOH + 0.1% Formic acid		
**Solvent B**	95% MeOH, 5% Water + 0.1% Formic acid		
**Gradient**	**Time (min)**	**%A**	**%B**
	0	95	5
	0.3	95	5
	2.2	5	95
	2.6	5	95
	2.61	95	5
	2.8	95	5
**Flow**	0.6 ml/min		
**Run Time**	2.8 min, use a divert valve for initial 0.5 minutes		

Supplementary Table 2: Clinical and molecular features of CRC PDX models

Specimen ID	Colon or rectal	Age at consent	Primary or metastatic	Previous chemotherapy/ treatment?	Stage	KRAS	NRAS	PIK3CA	BRAF	ATM	TP53
CRC-001	Colon	69	Primary	FOLFOX and bevacizumab	IV	Mut	WT	WT	WT	WT	WT
CRC-010	Rectal	52	Primary	No	III	WT	WT	WT	WT	WT	Mut
CRC-026	Colon	48	Metastatic	FOLFOX and bevacizumab	IV	WT	Mut	WT	WT	WT	WT
CRC-042	Rectal	73	Primary	FOLFIRI, Apomab, Bevacizumb	II	Mut	WT	Mut (3' UTR)	WT	WT	Mut
CRC-098	R Colon	50	Primary and Liver Met	No	IV	Mut	WT	Mut	WT	WT	Mut
CRC-102	Rectal-Sigmoid	55	Metastatic	FOLFOX	IV	Mut	WT	WT	WT	WT	Mut
CRC-108	Sigmoid	44	Metastatic	Capecitabine, Oxaliplatin and Avastin	IV	Mut	WT	WT	WT	WT	WT
CRC-125	Rectal	58	Metastatic	Yes- but don’t know chemo regimen	IV	WT	WT	WT	WT	WT	Mut
